# Eco-friendly optical sensor for precise detection of gold ions in diverse matrices through the integration of β-2-hydroxybenzyl-3-methoxy-2-hydroxyazastyrene in a PVC membrane

**DOI:** 10.1007/s00216-024-05324-7

**Published:** 2024-05-29

**Authors:** Mohamed Hemdan, Mohamed A. Ali, Alaa S. Amin

**Affiliations:** 1 School of Biotechnology, Badr University in Cairo (BUC), Badr City, Cairo, 11829 Egypt; 2 Chemistry Department, Faculty of Science, Benha University, Benha, Egypt

**Keywords:** Optical sensor, Azastyrene Schiff bases, Gold determination, Poly vinyl chloride membrane, Colorimetry, Environmental analysis

## Abstract

An environmentally conscious methodology is investigated for the precise and discerning identification of trace concentrations of gold ions in diverse matrices. A novel optical sensor membrane is proposed for the determination of Au^3+^ ions, utilizing the immobilization of β-2-hydroxybenzyl-3-methoxy-2-hydroxyazastyrene (HMHS) entrapped in polyvinyl chloride (PVC). The sensor incorporates sodium tetraphenylborate (Na-TPB) as the ionic additive and dibutyl phthalate (DBP) as a plasticizer. Under optimal conditions, the suggested sensor exhibits a linear calibration response to Au^3+^ ions within a concentration range of 5.0 to 165 ng mL^−1^. Detection and quantification limits are specified as 1.5 and 4.8 ng mL^−1^, respectively, with a rapid response time of 5.0 min. Upon presentation, this optical sensor not only affirms high reproducibility, stability, and an extended operational lifespan but also showcases exceptional selectivity for Au^3+^ ions. Notably, no discernible interference is observed when assessing the potential influence of other cations and anions on Au^3+^ ion detection. The adaptability of this optical sensor is validated through its successful application in determining Au^3+^ ion concentrations across various sample types, including water, environmental, cosmetics, and soil matrices.

**Graphical Abstract:**

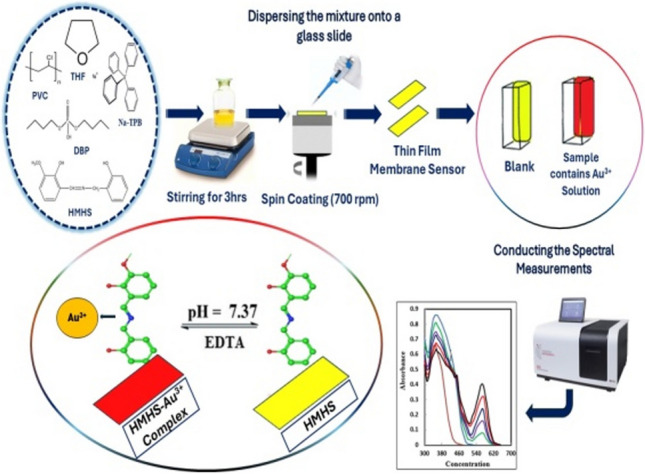

## Introduction

Gold is among the Earth's elements found in very limited natural quantities. As one of the key noble metals, it holds significant importance in various industrial and economic applications [1, 2]. Electronic waste, particularly from devices like cell phones and personal computers, represents a potential resource as they contain Au^3+^. Notably, cellular phones contain approximately 200 g of Au(III) per ton of scrap, a considerably higher concentration compared to Au(III) content in gold ores, which typically ranges from 10 to 30 g of gold per ton of ore [3–5].

Gold, an exceptionally precious metal, finds extensive applications across various domains such as aerospace, nuclear power, petrochemicals, electronics, information technology, metallurgy, electrical engineering, medicine, and jewelry [2, 6–11]. Furthermore, Au(III) levels are measured at 0.20 µg L^−1^ in river water and 0.05 µg L^−1^ in sea water [12–14]. The detection of Au^3+^ ions in environmental, geological, and metallurgical substances poses a formidable challenge due to the considerable interference from matrix components and the comparatively low concentrations of Au^3+^, frequently falling below the detection threshold of instrumental techniques.

Various analytical methods, including atomic absorption spectrometry [15], inductively coupled plasma atomic emission spectrometry (ICP-AES) [16], flame atomic absorption spectrometry (FAAS) [17, 18], graphite furnace atomic absorption spectrometry (GF-AAS) [19], electrothermal atomic absorption spectrometry (ETAAS) [20], microwave plasma atomic emission spectrometry [21], inductively coupled plasma mass spectrometry (ICP-MS) [22], spectrophotometric techniques [23, 24], fluorometric detection [25], voltammetric approaches [26], potentiometric titration [27], and neutron activation analysis (NAA) [28], have been employed to ascertain gold levels in diverse samples. However, the fundamental hurdle arises from the consistently meager concentration of gold, rendering the direct utilization of these analytical techniques unfeasible.

The employment of optical chemical sensors has experienced significant expansion over the past three decades, driven by their beneficial characteristics such as straightforward fabrication, exceptional selectivity and sensitivity, and cost-effectiveness. These optical chemical sensors, commonly known as optodes or optrodes, have become valuable instruments for evaluating analytes, depending on the careful selection of economical, effective, and insoluble chelating agents [29]. Transduction methods in optical sensors encompass the conversion of absorbance measurements into corresponding signals conveying information about the analyte. Optical absorption [30], fluorescence [31], and reflectance [32] stand out as extensively utilized techniques in optical chemical sensors. These approaches enhance the versatility and efficacy of optodes, rendering them selective and sensitive instruments for analytical purposes.

In most optodes, a substance is enclosed within a solid framework, usually in the form of a slim layer on a see-through base, like glass. Three frequently employed strategies for immobilization include chemical fixation on a suitable support [33], physical entrapment within polymeric matrices [34, 35], and adsorption onto the surface of the support material [36, 37], with the latter being the simplest approach. The substances integrated into the sensor play a vital role in extracting analytes into the sensing material and triggering an optical signal that mirrors alterations in analyte concentration. A prevalent approach in many established optodes involves color complexation reactions between analytes and an immobilized ligand [38–45]. This method contributes to the functionality of the sensor, allowing for the detection and quantification of analytes through observable changes in color.

Based on our comprehensive literature review, β-2-hydroxybenzyl-3-methoxy-2-hydroxyazastyrene (HMHS) has not been previously employed as a sensing agent. To establish a specialized optical sensor capable of spectrophotometrically detecting Au3 + in real aqueous sample solutions, HMHS is covalently immobilized onto a PVC membrane, serving as an efficient ionophore with nitrogen and oxygen donor atoms. The main goal of this research is to effectively incorporate HMHS into a plasticized PVC film, establishing an innovative optical sensor distinguished by outstanding selectivity and sensitivity. This sensor aims to facilitate the swift evaluation of Au^3+^ ions in biological and environmental samples.

## Materials and methods

### Chemicals and reagents

**Scheme 1:**
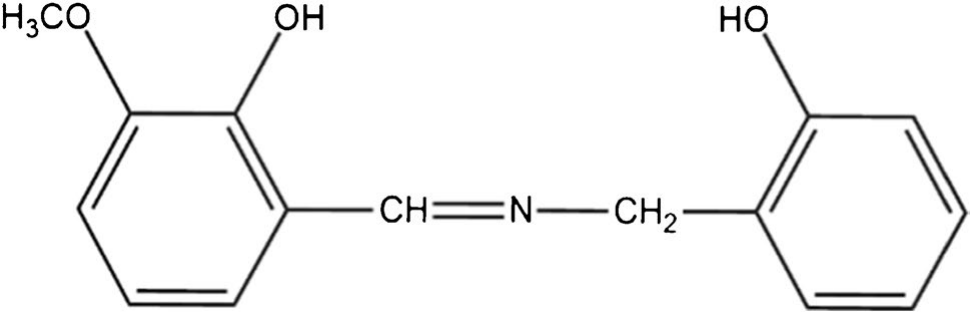
The chemical structure of the synthesized HMHS reagent

Every chemical utilized was of analytical reagent quality procured from Merck (Darmstadt, Germany) and was employed without additional purification. In the synthesis of the membrane, high-molecular-weight polyvinyl chloride (PVC), recently distilled tetrahydrofuran (THF), and dibutyl phthalate (DBP) were utilized. Sodium tetraphenylborate (Na-TPB) functioned as a membrane additive. The nitrate salts of the metal ions under investigation {obtained from Merck (Darmstadt, Germany)} were employed to create their respective stock solutions in deionized and doubly distilled water. A 1 × 10^−3^ M Au^3+^ stock solution was created by dissolving a predetermined amount of NaAuCl_4_·H_2_O {obtained from Merck (Darmstadt, Germany)} in a sufficient volume of deionized water, followed by subsequent volumetric standardization [46]. Working solutions with reduced concentrations were prepared through the dilution of the stock solution with water. The synthesis of β-2-hydroxybenzyl-3-methoxy-2-hydroxyazastyrene (HMHS) (Scheme 1) followed the procedure described earlier [47].

To examine the influence of diverse ions, solutions containing various metals were generated through the dissolution of predetermined amounts of their salts in either distilled water or diluted hydrochloric acid. Solutions of anions were formed by dissolving corresponding alkali metal salts in water. Buffer solutions with a universal pH range of 2.56–12.51 were precisely crafted using the Britton and Robinson method [48].

### Apparatus

All flame atomic absorption spectrometry (FAAS) measurements were conducted using the Perkin Elmer AAnalyst 300 model (Tokyo, Japan). Minor volumes of Au^3+^ ions were introduced into the cell via a Hamilton syringe (10 μL). The thickness of sensor was determined using a digital microscope (Ray Vision Y 103) coupled with a video camera (JVC TK-C 751 EG) and a digital micrometer (Mitutoyo, Japan) with an accuracy of ± 0.001 mm. The recording of spectra and absorbance measurements was carried out using a UV–vis spectrophotometer, model V 53 from JASCO (Tokyo, Japan). Absorbance readings were performed in a quartz cuvette by placing samples of optical membrane sensors (3.0 cm × 1.0 cm). The absorbance values of the optical membrane sensor samples were measured in reference to both air and a blank sensor sample. For pH analysis of solutions, an Orion Research model 601 A/digital ionalyzer pH meter was utilized. All experiments were carried out under ambient conditions at room temperature (25 ± 2 °C).

### Crafting the membrane for the optical sensor

The membrane fabrication involved meticulous blending of specific quantities of active components. Precisely, 30 mg of PVC, 60 mg of DBP, 4.0 mg of HMHS, and 6.0 mg of Na-TPB were completely dissolved in 5.0 mL of THF. Subsequently, 100 μL of the THF mixture was dispensed onto a pre-cleaned glass plate. After the cleaning process, where pure THF was used to eliminate organic impurities, 100 µL of the solution was applied to the glass plate. Measuring 1 mm × 9 mm × 50 mm, the glass plate was positioned in a spin-on device for a two-minute spin-on process at 700 rpm. Following this, the membrane air-dried naturally for ten minutes, completing the fabrication. The ideal membrane attained a thickness of about 5.0–7.0 μm. Throughout all investigations, the control membrane replicated the composition of the proposed membranes, except for the absence of HMHS.

### General procedure

The optical sensor membrane was inserted into the spectrophotometer cell, which contained 2.5 mL of a universal buffer at a pH of 7.37. Following this, a specified quantity of Au^3+^ ion solution (100 μL of 1.0 μg mL^−1^) was introduced into the cells and thoroughly mixed. After allowing for a 5.0-min equilibration period, the absorption spectrum was recorded across the wavelength range of 300–700 nm at 10 mm intervals, using a reference blank membrane. The measurement was conducted relative to a blank membrane, prepared in a similar manner but lacking Au^3+^, serving as a comparative reference.

### Analysis of Au^3+^ in real water samples

Water samples underwent filtration using Whatman filter paper no. 1 and no. 42, followed by the addition of varied amounts of Au^3+^. The proposed sensor membrane was placed into the treated water samples, adjusting the pH to 7.37 with a universal buffer, and Au^3+^ spiked water samples for 10 min. After preparation, the sample was moved to a quartz cell, and the sensor membrane was positioned diagonally within. The Au^3+^ concentration was determined using the calibration graph. For validation, FAAS was used for comparative analysis to confirm the accuracy of Au^3+^ concentration in water samples after preconcentration process [18].

### Analysis of Au^3+^ in soil samples

A 2.0 g sample of soil, sediment, and ore underwent digestion utilizing 2.0 mL of HNO_3_ (65%) and 6.0 mL of HCl (37%) in a microwave digestion system. Digestion parameters included 2 min at 0 W, 2 min at 250 W, 5 min at 400 W, 6 min at 250 W, 8 min at 550 W, and venting for 8.0 min [49]. The resulting sediment was thinned to a concluding volume of 50 mL with purified water. A control digestion employing the identical procedure was executed. Afterward, the outlined method was employed on the ultimate solutions, and the gold concentration was ascertained after preconcentration process using flame atomic absorption spectrometry (FAAS) [18] for comparative assessment.

### Analysis of Au^3+^ in cosmetics

A 0.25 g sample of cosmetics underwent digestion using 3.0 mL of concentrated HCl and 1.0 mL of concentrated HNO_3_ in a microwave oven (23–43 atm; 50% microwave power; 7.0 min heating time). Following digestion, the samples were thinned to a concluding volume of 25 mL with double-distilled water, and the resultant solutions were tuned to a pH of 7.37 using a universal buffer solution. These solutions were subsequently transferred to a quartz cell measuring 1.0 cm in length, and the sensor membrane was diagonally positioned within the quartz cell. The determination of Au^3+^ content in the cosmetic sample was carried out utilizing the calibration graph and FAAS after preconcentration process for the treated sample [18].

### Analysis of Au^3+^ in computer circuit

For assessing gold in computer circuit board remnants acquired from the nearby computer market, a suggested technique was utilized. Ideal parameters were determined for applying the suggested sensor membrane approach to diverse computer circuit board remnants (amounting to 100 g). The preparation of the scraps involved a three-step process. Initially, the scraps were treated with a (1:2) mixture of concentrated HNO_3_ and water (200 mL) at 70 °C for 3.0 h to dissolve base metals. Afterward, the liquid and solid components were isolated, and the solid material underwent rinsing with deionized water. In the final step, the solid substance was subjected to 200 mL of aqua regia for 3.0 h at 25 ± 2.0 °C, causing the dissolution of all metallic constituents. Subsequently, the pH of the solution was regulated to 0.5, and it underwent dilution to achieve a final volume of 500 mL [50]. The resultant solution underwent an additional 2000-fold dilution with pH 7.37 universal buffer solution and was then transferred into a 1.0 cm quartz cell. Placed diagonally within the quartz cell, the sensor membrane facilitated the measurement of absorbance at 70 °C with a λ_max_ of 568 nm. The determination of Au^3+^ concentration in computer circuit board samples followed, utilizing a calibration graph constructed from the absorbance measurements. The gold concentration was ascertained after preconcentration process using flame atomic absorption spectrometry (FAAS) [18] for comparative assessment.

## Results and discussions

### Spectral analysis

**Fig. 1:**
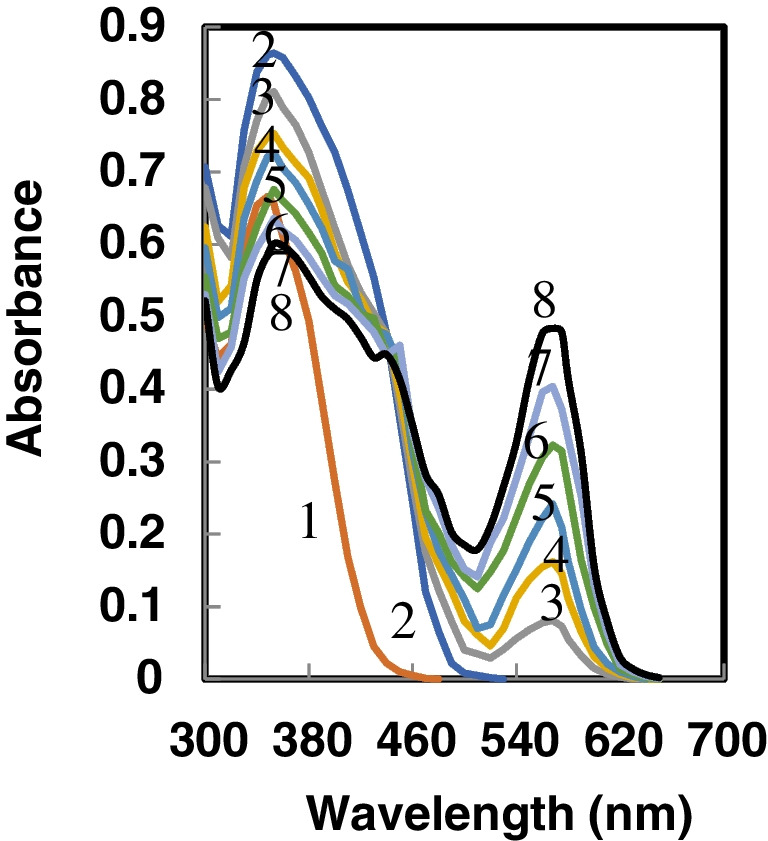
Absorbance spectra of 1-HMHS in solution; 2-  HMSH sensor membrane; and 3–8 membrane in the presence of increasing [Au^3+^] ion 25,50,75,100,125, and 150 ng mL^−1^

Figure 1 illustrates absorption spectra for both unbound Au^3+^ ions and HMHS embedded in the membrane. These spectral readings were captured following stabilization in a buffer solution with a pH of 7.37, encompassing various concentrations of Au^3+^ ions. The optode's spectral features revealed two peaks at 353 and 568 nm. The noted decline in the peak of absorption at 353 nm and the simultaneous rise in the absorption peak at 568 nm as the concentration of Au^3+^ ions increases can be attributed to the incorporation of Au^3+^ ions into the membrane, followed by chelation and complexation processes facilitated by the HMHS ionophore.

Indeed, the operational process of the optode is assumed to depend on the complexation between HMHS and Au^3+^. It is notable that the absorption patterns of HMHS show a bathochromic shift when compared to the patterns observed when the molecule is dissolved in THF (353 nm instead of 348 nm). This insight indicates that the structured arrangement of the immobilized indicators is probably more planar than its soluble form. The choice of the 353 nm wavelength for subsequent analyses was determined by its proven heightened selectivity and sensitivity.

### Impact of varying HMHS amounts on optode response

**Fig. 2:**
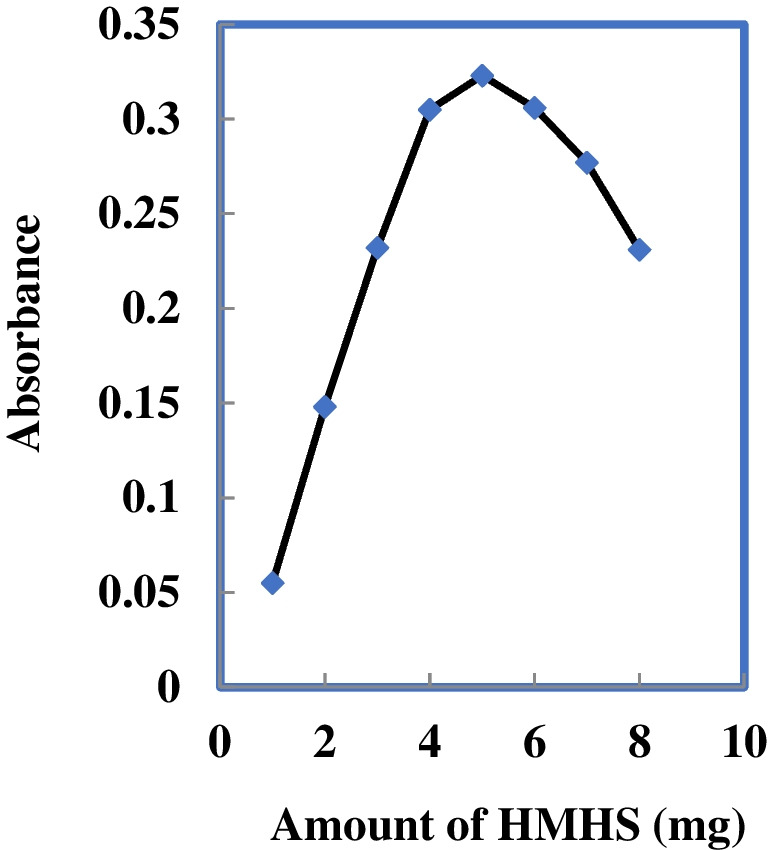
Effect of amount of HMHS on the response of the proposed sensor (in the presence of 100 ng mL^−1^ Au.^3+^)

The investigation focused on the impact of varying the ionophore quantity within the range of 1.0–8.0 mg while maintaining a constant Au^3+^ concentration of 100 ng mL^−1^, and the findings are depicted in Fig. 2. The augmentation of the HMHS ionophore amount up to 4.0 mg was noted to significantly enhance sensitivity, attributed to the increased intensity of complexation between HMHS and Au^3+^ ions. However, beyond 4.0 mg, a significant decline in sensitivity occurred, possibly because of the creation of a charged complex leading to back extraction into an aqueous solution or membrane saturation. On the flip side, with lower amounts of HMHS, the likelihood of an inadequate number of reactive sites probably contributed to a reduction in complexation and Au^3+^ ion mass transfer. This led to reduced sensitivity and extraction.

### Impact of Na-TPB quantity

**Fig. 3:**
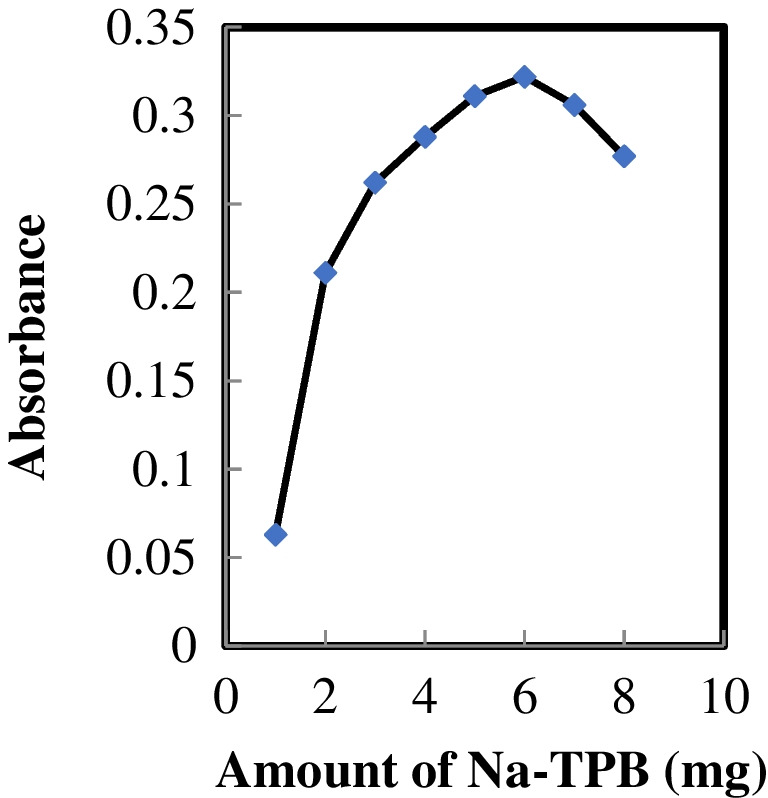
Effect of amount of Na-TPB on the response of the proposed optode (in the presence of 190 ng mL^−1^ Au.^3+^ ions)

The selectivity and sensitivity achieved with a specific ionophore (reagent) are well-acknowledged to be heavily influenced by the composition of the membrane as well as the properties of the solvent mediator and additives [51, 52]. Hence, an investigation was carried out to assess how the quantity of Na-TPB, functioning as a lipophilic additive (anionic site), influences the response characteristics of the optode. As depicted in Fig. 3, augmenting the quantity of Na-TPB up to 6.0 mg contributed to an enhancement in the sensor's response. Further increases in Na-TPB did not have a substantial effect on the response. In contrast, a reduced quantity of the additive led to diminished perm-selectivity and impeded Au^3+^ ion mass transfer to the surface of the membrane, resulting in a a noteworthy reduction in sensitivity. Consequently, the recommendation for subsequent studies is to utilize 6.0 mg of Na-TPB.

### Impact of pH on sensor response

**Fig. 4:**
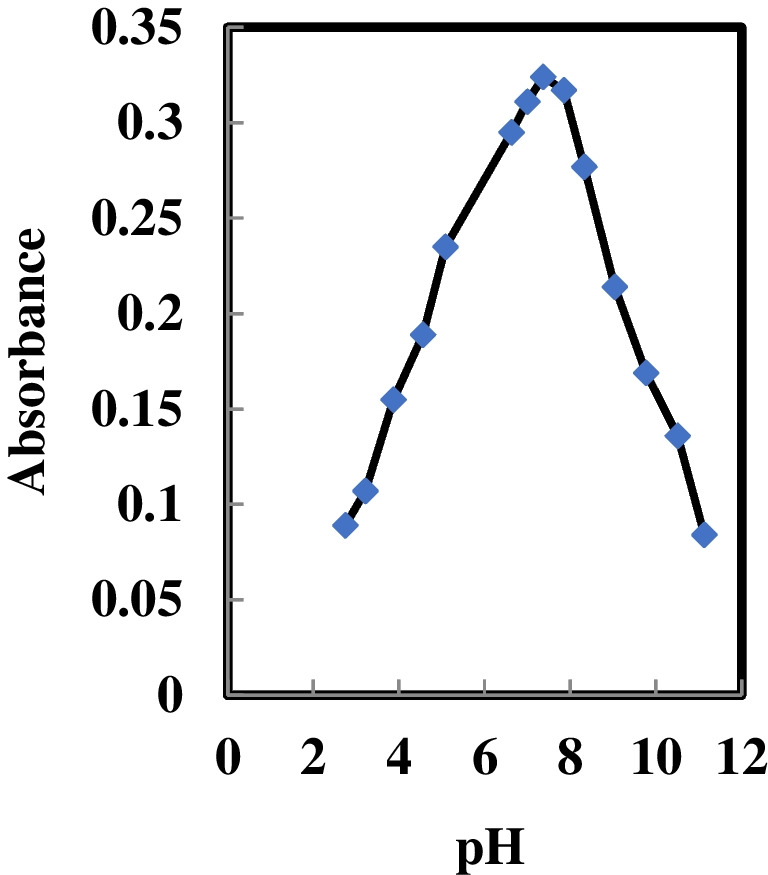
Effect of pH on the response of the proposed sensor (in the presence of 100 ng mL^−1^ Au.^3+^ ions)

The integration of Au^3+^ ions into the membrane phase corresponds to the release of hydrogen ions from the membrane into the solution. Accordingly, the membrane's reaction to Au^3+^ is markedly influenced by acidity, as the signal intricately relates to the concentrations of Au^3+^ ions and hydrogen ions [36]. In Fig. 4, the reliance of the suggested sensor's reaction is depicted across diverse acidity levels, with optimal sensitivity observed at an acidity of 7.37. At elevated acidity levels, a substantial reduction in sensitivity was observed, likely attributed to the creation of insoluble species like Au (OH)_3_ or partially soluble [Au(OH)_2_H_2_O]^+^, or other hydroxo complexes.

On the contrary, at increased pH levels, the deprotonation of an HMHS functional group initiates a reversed dispersion of ionophores, constraining the diffusion and migration of Au^3+^ ions to the surface of membrane. In contrast, at low values of pH, potential protonation of HMHS ionophores and potential hydrolysis of the aqueous solution (i.e., Au^3+^ ions) lead to a notable decrease in the complexation and extraction of Au^3+^ ions within the sensor membrane formulation. These mechanisms collectively result in a substantial reduction in the selectivity and sensitivity of the sensor.

### Dynamic range

**Fig. 5:**
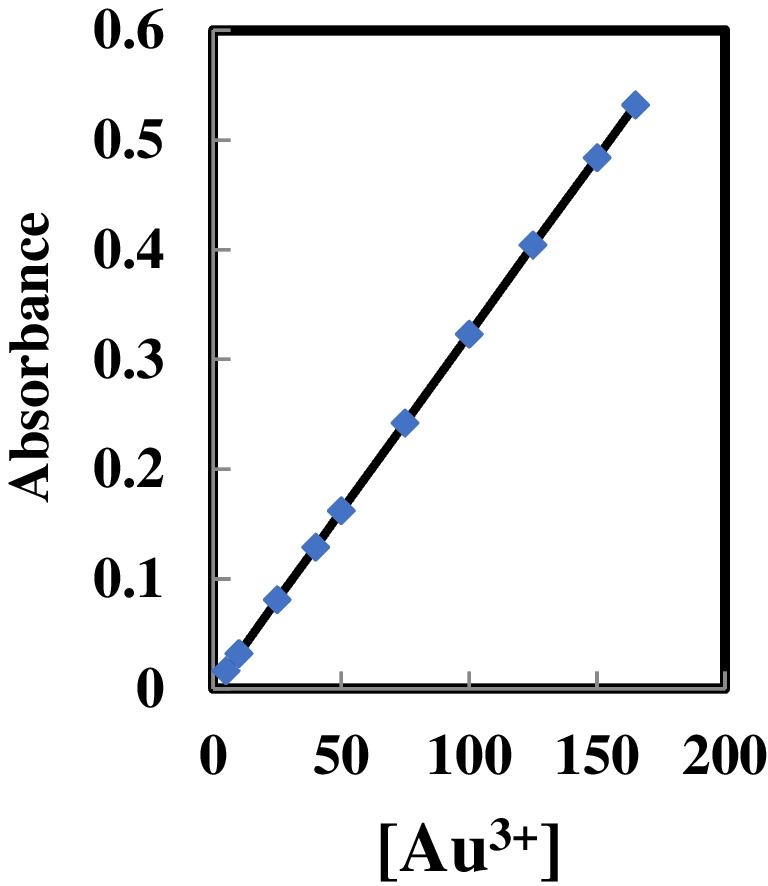
Calibration curve of the membrane at 568 nm vs. [Au^3+^] ions

In Fig. 5, the calibration plot illustrates the signals of absorption of the sensor membrane under conditions previously fine-tuned across different concentrations of Au^3+^ ions. The signal is determined by absorbance, representing the difference in absorbance between the membrane with HMHS in the presence and absence of Au^3+^ ions. It is evident from the graph that the calibration is linear within the concentration range of 5.0 to 165 ng mL^−1^. Detection and quantification limits were calculated using a well-established equation that incorporates the calibration graph’s slope and the standard deviation of blank membranes and solutions [53]. According to this analysis, the achieved limits were 1.5 ng mL^−1^ and 4.8 ng mL^−1^, respectively. These results demonstrate the effectiveness of the procedure in identifying trace levels of Au^3+^ ions. The implication is that using an affordable and readily available instrument, such as a UV–vis spectrophotometer in conjunction with an optode, allows for the precise and accurate determination of low concentrations of Au^3+^ ions.

### Response time

A pivotal analytical characteristic of any sensor membrane is its response time, defined as the minimum duration needed to achieve approximately 95% of the ultimate equilibrium response. In this investigation, under optimal conditions, the sensor membrane was found to achieve 95% of the maximum signal within 5.0 min, depending on the concentration of Au^3+^ ions. The sensitivity of the suggested sensor is impacted by the time necessary for the analyte to diffuse from the solution bulk to the membrane interface and engage with the HMHS ionophore. Significantly, the response time exhibits an inverse correlation with the initial concentrations of Au^3+^ ions. With an increase in Au^3+^ ion concentration from 25 to 150 ng mL^−1^, the response time notably prolonged from 3.0 min to 5.0 min. The temporal progression of the intensity of absorption of the membrane at 568 nm is illustrated in Fig. 7. Typically, the response time is shorter in concentrated solutions as opposed to dilute solutions.

### Regeneration and lifetime

**Fig. 6:**
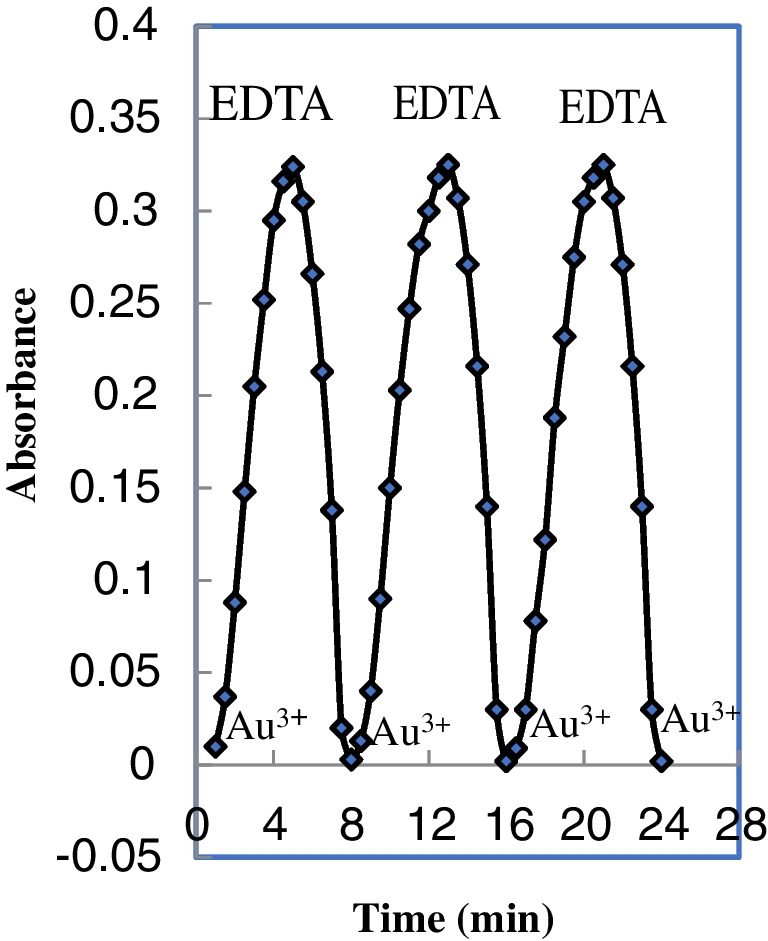
Repeatability of the sensor membran exposed 100 ng mL^−1^ Au^3+^ and 0.1 M EDTA

**Fig. 7:**
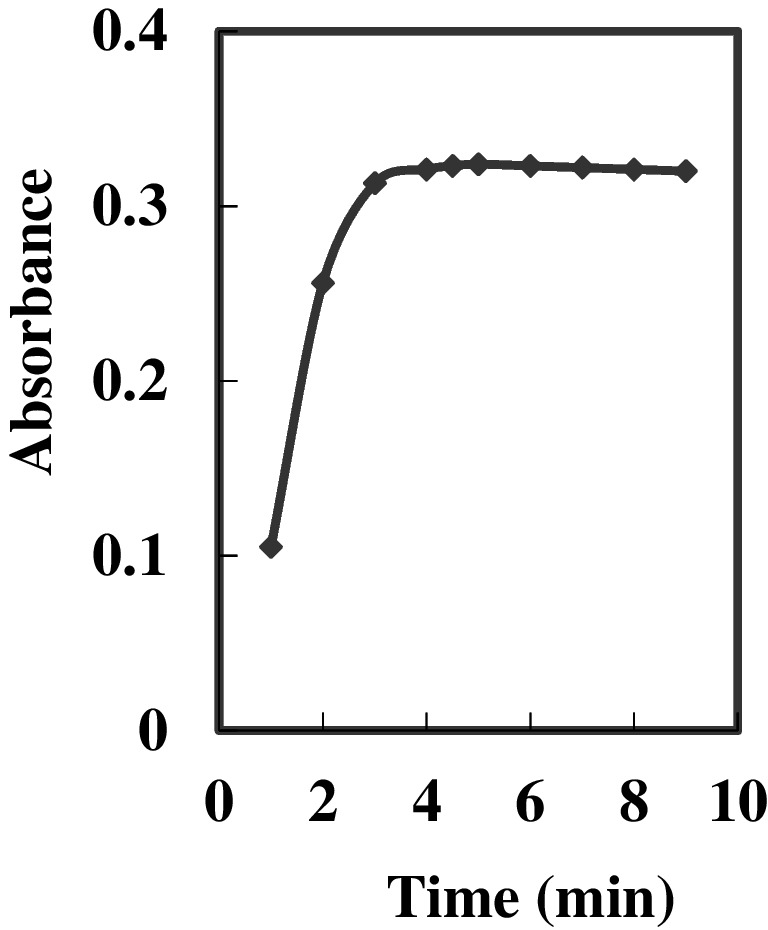
Response time of the proposed optode

Regenerating reagents such as ethylenediamine, thiourea, SCN, sulfosalicylic acid, EDTA, and HNO_3_ and HCl acids were studied. However, there was no additional improvement observed in the optode's reversibility after partial and lengthy exposure to ethylenediamine, thiourea, SCN, sulfosalicylic acid, HNO_3_ and HCl. Among the reagents tested, EDTA, proved to be the optimal choice, as it had a quick regeneration period of about three min. An ideal sensor should regenerate entirely for repeated use in a short duration (Fig. 6). The sensor can be regenerated and reused by exposure to an EDTA solution with a pH of 7.0. A quick regeneration time of less than three min was achieved using a 0.25 mL of 0.1 M solution of EDTA. The on use durability of the sensor phase was achieved by subsequently placing the membrane in Au^3+^ solutions and regenerating. After regeneration and for the next Au^3+^ concentration measurement, the optode should be putted in buffer for 1.0–2.0 min (Fig. 7).

To evaluate the durability of the suggested sensor, the membrane was preserved in air, ethanol, or deionized water and absorbance measurements were taken at consistent intervals (every 6.0 h). The criteria for determining the sensor's lifetime included monitoring changes in absorbance, alterations in the linearity range, shifts in the calibration curve slope, and variations in detection and quantification limits. Measurements based on these parameters indicate a consistent response of the sensor membrane for a minimum of one week, with no notable changes observed.

### Effect of temperature

The influence of temperature on the sensor's performance was examined by capturing absorption spectra at different temperatures ranging from 25 to 60 °C, specifically at 568 nm. With an increase in the temperature of the Au^3+^ sample, a reduction in absorbance at 568 nm was observed. This decrease is attributed to thermal quenching, associated with the heightened lattice vibrations of ions [54], and a concurrent decline in the creation of the complex with the membrane. Beyond a temperature of ≥ 60 °C, there was no detectable alteration in absorbance, signifying the lack of complex formation between Au^3+^ and HMHS. The ideal temperature for achieving highly selective and sensitive outcomes was determined to be 25 ± 2.0 °C.

### Impact of stirring

The response of the developed sensor is notably affected by external stirring of the Au3 + solution. Using a magnetic stirrer with a maximum speed of 400 rpm for convective diffusion during the spin-on process, agitating the Au3 + solution resulted in approximately a six-fold increase in enrichment compared to when the solution was not agitated. This enhancement can be attributed to the facilitated movement of Au3 + ions towards the immobilized HMHS during stirring. The stirring action accelerates the diffusion of Au3 + ions through the sensor membrane towards HMHS, thereby expediting the reaction between Au3 + ions and HMHS. In contrast, under non-stirring conditions, the diffusion of Au3 + ions across the sensor membrane relies solely on the concentration gradient [55]. The stark contrast in optode response underscores the significant impact of stirring in the experimental setup.

### Membrane thickness

The mean thickness of the manufactured PIM was determined to be 0.570 ± 0.002 mm. During the experiments, the thickness of the sensor membrane was precisely measured using a digital microscope (Ray Vision Y 103) connected to a video camera (JVC TK-C 751EG). This measured thickness is deemed suitable for ion mobility in the Au^3+^-HMHS complex reaction. The membrane thickness falls within a favorable range, being neither excessively thick (> 1.000 mm) nor excessively thin (< 0.005 mm). This thickness is deemed reasonable for serving as a transducer in the sensor membrane based on the principle of co-extraction [56].

### Stoichiometry of Au^3+^- HMHS complex

The assessment of the stoichiometry of the Au^3+^-HMHS complex was performed utilizing the mole ratio method and Job's method. HMHS exhibits its highest absorption at 353 nm, whereas the Au^3+^-HMHS complex demonstrates its maximum absorption at 568 nm. Both techniques were applied at a wavelength of 568 nm, corresponding to the complex's peak absorption. Results from both Job's method [57] and the mole ratio method indicate a 1:2 stoichiometry for the formed complex (Au-HMHS).

### Sensor selectivity

A crucial aspect of an optical sensor is its relative response concerning the primary ion in comparison to the concurrent ions present in the solution [58]. Interfering factors encompass anions and cations that may react with the HMHS ionophore in the membrane sensor or species capable of reacting with Au^3+^ ions, thereby impeding migration and diffusion efficiency. The tolerance limit was established as the concentration that induces an error exceeding ± 5.0% in the signal associated with a constant concentration of Au^3+^ ions, and is referred to as the tolerance limit [59, 60]. In this context, the membrane’s absorbance was measured after and before introducing a constant concentration of interference ions into a known amount of the Au^3+^ ion solution, with the results presented in Table 1.

**Table 1:** Tolerance ratio (TR = ion/Pd^2+^ mass ratio) for various interfering ions in the determination of 100 ng mL^−1^ of Au^3+^

Ion	TR	Ion	TR
Na^+^, K^+^, Acetate	12,000	Al^3+^, Fe^3+^, CO_3_^2−^	3000
Ca^2+^, Mg^2+^, Succinate	10,000	Fe^2+^, Oxalate	2500
Li^+^, Tl^+^, Citrate	8500	Cu^2+^, Ni^2+^, SO_4_^2−^	2000
Sr^2+^, Ba^2+^, NO_2_^−^, Cl^−^	7000	Hg^2+^, Co^2+^, HCO_3_^2−^	1500
Ge^4+^, Ti^4+^, NO_3_^−^	6000	Sn^4+^, Sn^2+^, Cd^2+^	1250
Bi^2+^, Mn^2+^, IO_3_^−^	5000	Zn^2+^, Pb^2+^, SCN^−^	1000
Zr^4+^, Cr^6+^, S_2_O_3_^2−^	4500	Au^3+^, La^3+^, Cr^3+^	500
Mo^6+^, W^6+^, Br^−^	4000	Se^4+^, Te^**4+**^, Pt^4+^	200
Th^4+^, UO_2_^2+^, B_4_O_7_^2−^	3500	Cu^2+^	75

The results obtained confirm the exceptional selectivity of the sensor membrane towards Au^3+^ ions at λ_max_ = 568 nm, demonstrating negligible interference from coexisting ions, except for Cu^2+^ ions, which do not interfere upto concentrations below 75 mass ratios. Above this concentration, it presents a challenge in the determination of Au^3+^ ions compared to other investigated cations, potentially affecting the accuracy of the analysis. This remarkable selectivity can be attributed to the presence of nitrogen atoms with soft borderlines and the NH group. These components, through distinct mechanisms, exhibit a strong affinity for binding Au^3+^ ions, recognized as the most reactive and the strongest ions in the Irvin Williams series, primarily via metal-ions' dipole interaction and soft–soft interaction. Additionally, the high resonance contribution in the HMHS structure enhances its reactivity in binding metal ions. The formation constants of HMHS complexes presented in Table 3 further confirm the selectivity of the developed sensor. Consequently, it appears viable to utilize the developed sensor for practical Au^3+^ assays.

### Repeatability and reproducibility

The sensor membrane's response repeatability at 568 nm was assessed using a single membrane, with six consecutive measurements conducted at a concentration of 100 ng mL^−1^ of Au^3+^ ions. The determined relative standard deviation (RSD) for these measurements was below 2.25%. To evaluate reproducibility, a similar approach was applied to six distinct membranes at various time intervals. The RSD for the measurement of 100 ng mL^−1^ of Au^3+^ ions was recorded at 1.94%. It is evident that the sensor presented in this study exhibits notable precision, both in terms of reproducibility and repeatability, making it well-suited for diverse applications in varying conditions.

### Analytical applications

The effectiveness and legitimacy of the sensor membrane presented for estimating Au^3+^ ion concentration were affirmed through the examination of actual samples, specifically water samples, and the outcomes are detailed in Table 2. The results demonstrate noteworthy recovery rates and reasonable RSD for these analyses, validating its suitability for precise and consistent monitoring of Au^3+^ ion levels. The analytical characteristics of the fine-tuned sensor membrane, encompassing the regression equation, linearity range, standard deviation, and limits of detection and quantification for Au^3+^ determination, are succinctly outlined in Table 2. The outcomes obtained serve as robust indicators of the effectiveness of the suggested approach in achieving sensitive, selective, and effective monitoring of Au^3+^ ion content across diverse water samples, even those with complex matrices and at low concentrations. The apparent success of the proposed ionophore (HMHS), renowned for its high selectivity, underscores its efficacy as a proficient collector for the effective preconcentration and accumulation of Au^3+^ ions within the membrane.

**Table 2:** Utilization of the established sensor for the analysis of Au^3+^ ions in water and soil samples

Samples	Added [ng mL^−1^]	Found^a^ [ng mL^−1^]		Recovery (%)	t-test^b^	F-value^b^
		Sensor	FAAS [18]			
Tap water	–	–	–			
	30.0	29.5 ± 0.08	31.0 ± 0.77	98.33	1.57	
	60.0	59.4 ± 0.13	61.7 ± 1.05	99.00		3.77
Mineral water	–	–	–			
	40.0	40.5 ± 0.11	38.9 ± 1.20	101.25	1.76	
	80.0	78.9 ± 0.18	81.6 ± 0.95	98.63		3.93
Underground water	–	–	–			
	70.0	71.4 ± 0.09	68.7 ± 1.05	101.93	2.22	
	140	138.0 ± 0.10	142.2 ± 1.30	98.57		4.05
Rain	–	–	–			
water	75.0	76.5 ± 0.07	73.70 ± 0.80	102.00	1.88	
	150	147.8 ± 0.12	153.5 ± 1.00	98.53		3.93
River water	–	5.5 ± 0.06	5.5 ± 1.10			
	65.0	63.7 ± 0.08	63.0 ± 1.25	98.00	1.62	
	130	132.3 ± 0.13	137.2 ± 0.75	101.77		3.73
Sea water	–	3.0 ± 0.08	3.0 ± 1.15	–		
	45.0	49.1 ± 0.16	47.0 ± 0.95	102.29		4.04
	90	91.3 ± 0.20	94.5 ± 0.85	98.17	1.97	
Pond water	–	7.5 ± 0.25	7.5 ± 1.20	–		
	55.0	63.3 ± 0.21	60.2 ± 1.45	101.28		3.78
	110	114.7 ± 0.13	119.4 ± 0.95	97.62	2.23	
Industrial wastewater	–	15.2 ± 0.07	15.1 ± 1.05			
	35.0	51.5 ± 0.11	48.9 ± 1.30	102.59	2.08	
	70.0	83.7 ± 0.17	87.0 ± 1.55	98.25		3.75
Soil^c^	–	–	–			
	65.0	66.8 ± 0.14	63.7 ± 1.00	102.69	2.33	
	130	127.5 ± 0.19	131.8 ± 1.35	98.04		4.16
Sediment^c^	–	–	–			
	55.0	56.3 ± 0.11	53.8 ± 2.05	102.36	2.17	
	110	108.2 ± 0.13	113.1 ± 1.85	98.36		3.89
	–	–	–			
Mine^c^	75.0	76.7 ± 0.18	73.5 ± 0.19	102.26	1.95	
	150	151.9 ± 0.20	147.6 ± 0.19	101.27		3.65

^a^ Mean ± SD

^b^ Theoretical values for t- and F- values at 95% confidence level for five degrees of freedom are 2.57 and 5.05, respectively

^c^ µg kg^−1^

To validate both the practical and analytical reliability of the suggested sensor, soil samples were employed for determining Au^3+^ ions. The outcomes derived from the proposed procedure were juxtaposed with those acquired through Flame Atomic Absorption Spectroscopy (FAAS) after preconcentration process [18] of the examined sample, as presented in Table 2. A comparative analysis using the Student’s t-test, with a 95% confidence level, was conducted to assess the concordance between the two methods. By separately analyzing six replicate samples through the proposed sensor membrane and FAAS after preconcentration process [18] of the examined samples, the calculated t-value (t_calc_.) suggested that the methods did not exhibit significant differences, and the results were consistent within the confines of experimental error.

The developed methodology was implemented for the quantitative detection of minute quantities of gold in actual cosmetic matrices. Gold concentrations ranging from 25.6 to 43.5 ng g^−1^ were ascertained in five facial cosmetics, including creams, serums, and cream masks. The two face serums exhibited the highest gold concentrations at 43.5 and 33.6 ng g^−1^. The gold concentrations in two face creams and one face cream mask were found to be similar, ranging from 25.6 to 27.2 ng g^−1^. Notably, among the remaining eleven cosmetics, including cream mask (two), sunscreen cream (two), pads active mask (two), face cream (three), face serum gel (two), no gold was detected. This implies that either these cosmetics do not contain gold or its levels are below the Limit of Detection (LOD) of the proposed procedure. To verify the accuracy of the presented methodology, cosmetic samples were subjected to analysis using the standard addition method. The cosmetic samples underwent spiking with Au^3+^ concentrations ranging from 10 to 60 ng g^−1^. The achieved recoveries, falling between 98.61% and 101.98%, underscore the effectiveness of the described gold detection procedure in cosmetics, as detailed in Table 3. To assess the precision of the developed method, Flame Atomic Absorption Spectroscopy (FAAS) was utilized as a comparative technique for determining analyte concentrations after preconcentration process [18] in cosmetics samples. The F-value, calculated as the ratio of variances (s12/s22), was employed to test the significance of the difference between the variances of the two procedures. This test, assuming normal populations, yielded an F-value indicating no statistical difference in precision between the proposed sensor membrane and FAAS (Table 3). Subsequently, the Student’s t-test was employed to scrutinize the statistical differences in accuracy between the results obtained through the proposed procedure and the comparative technique. As evident from Table 3, the calculated t-values in each instance were below the critical value (t_crit_ = 2.57), affirming the absence of statistical differences in results. The congruence of FAAS results with detected analyte levels underscores the efficacy of the proposed procedure for nano-trace analysis of gold in cosmetic products.

**Table 3:** Determination of total gold concentration in cosmetics using the proposed sensor and FAAS procedures. The uncertainties correspond to one standard deviation (n = 5)

		Found Au^3+^ ng g^−1^			F-value^a^	T-test^a^
Cosmetics		Proposed procedure		FAAS [18]		
	Spiked	Total	Recovery %	Total		
Face cream	–	27.5 ± 1.58		27.1 ± 1.78	2.08	
	10	38.0 ± 1.34	101.33	36.4 ± 1.95		0.703
	20	57.1 ± 1.06	99.30	58.2 ± 2.08	2.32	
Face cream	–	27.2 ± 1.05	–	26.8 ± 1.13		0.95
	15	41.8 ± 1.22	99.05	43.5 ± 1.83	2.16	
	30	58.5 ± 1.47	102.27	55.7 ± 2.16		0.79
Face serum	–	43.5 ± 1.33	–	44.0 ± 1.38	2.93	
	20	64.0 ± 1.19	100.79	65.5 ± 1.93		0.65
	40	82.7 ± 1.52	99.04	86.4 ± 2.22	2.85	
Face serum	–	33.6 ± 1.21	–	34.0 ± 1.31		0.87
	30	64.5 ± 1.13	101.80	62.7 ± 2.37		
	60	92.3 ± 1.17	98.61	95.6 ± 2.12	1.22	
Face cream mask	–	25.6 ± 1.04	–	25.5 ± 1.15		0.94
	25	50.0 ± 1.37	98.81	52.3 ± 1.92	2.55	
	50	77.1 ± 1.60	101.98	83.3 ± 2.25		1.17

^a^ Theoretical values for t- and F- values at 95% confidence level for five degrees of freedom are 2.57 and 5.05, respectively

To assess the precision and applicability of the current method for analyzing computer circuit board samples, appropriate aliquots were dissolved as outlined above and subjected to the developed procedure under optimized conditions. To verify the reliability of the procedure, the gold concentration in the computer circuit board samples was concurrently measured using Flame Atomic Absorption Spectroscopy (FAAS). The outcomes presented in Table 4 demonstrate the successful application of the sensor membrane for determining gold levels in real environmental samples. The relative error calculated with the sensor was found to be 2.0% at a 95% confidence level with a sample size (n) of 6, indicating the high accuracy of the method.

**Table 4:** Analysis of Au^3+^ in computer circuit board samples through six repetitive measurements using the established sensor and FAAS

Au^3+^in sample^a^ (mg kg^−1^)	Added Au^3+^	Total Au^3+^	Present procedure	FAAS [18]	*RE* (%)	Recovery (%)	t-test^b^	F-value^b^
	(mg)	(mg kg^−1^)	(mg kg^−1^)	(mg kg^−1^)				
250	–	250	247.4 ± 0.21	253.5 ± 0.17	1.79	98.96	1.08	2.44
250	20	270	265.5 ± 0.63	274.2 ± 0.66	1.93	98.33	0.89	2.05
250	60	310	314.7 ± 0.65	316.9 ± 0.73	2.15	101.52	1.36	3.11
250	100	350	355.9 ± 0.77	347.3 ± 0.52	2.31	101.69	1.21	2.73

RE: Relative error

^a^ Computer circuit scrap

^b^ Theoretical values for t- and F- values at 95% confidence level for five degrees of freedom are 2.57 and 5.05, respectively

## Conclusions

In conclusion, the developed Au^3+^ ion-detecting sensor, utilizing a PVC membrane with β-2-hydroxybenzyl-3-methoxy-2-hydroxyazastyrene (HMHS) and sodium tetraphenylborate (Na-TPB), exhibits exceptional performance. Optimized parameters yield a linear calibration range of 5.0 to 165 ng mL^−1^, with detection and quantification limits at 1.5 and 4.8 ng mL^−1^, respectively. A rapid 5.0-min response time enhances practicality. The sensor demonstrates high reproducibility, stability, and selectivity for Au^3+^ ions. The membrane's reversible yellow-to-red color change adds visual detection and specificity. The sensor adapts well to diverse sample types (water, environmental, cosmetics, soil), showing superior performance without interference from other ions. Overall, this cost-effective and precise optical sensor, with its innovative membrane design and colorimetric response, contributes to gold determination technology. Its applications extend to environmental analysis and quality control across varied sample matrices, marking a significant advancement in optical sensing for Au^3+^ ions. To assess the precision of the developed method, FAAS [18] was utilized as a comparative technique for determining analyte concentrations after preconcentration process in cosmetics samples. The calculated t-test and F-value, were employed to test the accuracy and precision did not exceeded the theoretical values indicating that there is no significance differences between the variances of the two procedures.

## Data Availability

Data will be made available on request.

## References

[1] Fazli Y, Hassan J, Karbasi MH, Sarkouhi M (2022). A simple spectrophotometric method for determination of gold (III) in aqueous samples. Miner Eng.

[2] Pyrzynska K (2012). Sorbent materials for separation and preconcentration of gold in environmental and geological samples - A review. Anal Chim Acta.

[3] Ogata T, Nakano Y (2005). Mechanisms of gold recovery from aqueous solutions using a novel tannin gel adsorbent synthesized from natural condensed tannin. Water Res.

[4] Khoo KM, Ting YP (2001). Biosorption of gold by immobilized fungal biomass. Biochem Eng J.

[5] Syed S (2012). Recovery of gold from secondary sources—A review. Hydrometallurgy.

[6] Danscher G, Larsen A (2010). Effects of dissolucytotic gold ions on recovering brain lesions. Histochem Cell Biol.

[7] Sheoran V, Sheoran AS, Poonia P (2013). Phytomining of gold: a review. J Geochemical Explor.

[8] Chenghui W, Denghong W, Jue X, Lijuan Y, Lijun L, Shanbao L (2015). A preliminary review of metallogenic regularity of gold deposits in China. Acta Geol Sin Ed.

[9] Noel JG (2016). Review of the properties of gold material for MEMS membrane applications. IET Circuits, Devices Syst.

[10] Maduraiveeran G, Ramaraj R (2017). Gold nanoparticle-based sensing platform of hydrazine, sulfite, and nitrite for food safety and environmental monitoring. J Anal Sci Technol.

[11] Alim S, Vejayan J, Yusoff MM, Kafi AKM (2018). Recent uses of carbon nanotubes & gold nanoparticles in electrochemistry with application in biosensing: A review. Biosens Bioelectron.

[12] Zhang L, Li Z, Hu Z, Chang X (2011). Solid phase extraction of gold (III) on attapulgite modified with triocarbohydrazide prior to its determination in environmental samples by ICP-OES. Spectrochim Acta Part A Mol Biomol Spectrosc.

[13] Liu R, Liang P (2007). Determination of gold by nanometer titanium dioxide immobilized on silica gel packed microcolumn and flame atomic absorption spectrometry in geological and water samples. Anal Chim Acta.

[14] Liang P, Zhao E, Ding Q, Du D (2008). Multiwalled carbon nanotubes microcolumn preconcentration and determination of gold in geological and water samples by flame atomic absorption spectrometry. Spectrochim Acta Part B At Spectrosc.

[15] Giertyas CJ, Silva VES, de Oliveira MJ, Freire ES, Santos JCC, de Almeida RM, Meneghetti MR, Bortoluzzi JH (2022). Atomic absorption spectrometry as an alternative to determine the presence of gold nanoparticles on or in silica matrix. J Braz Chem Soc.

[16] Hassan J, Zari N, Tabar-Heydar K, Ahmadi SH (2016). Ion-association dispersive liquid–liquid microextraction of ultra-trace amount of gold in water samples using Aliquat 336 prior to inductively coupled plasma atomic emission spectrometry determination. J Anal Sci Technol.

[17] Duran A, Tuzen M, Soylak M (2015). Separation and enrichment of gold in water, geological and environmental samples by solid phase extraction on multiwalled carbon nanotubes prior to its determination by flame atomic absorption spectrometry. J AOAC Inter.

[18] Unsal YE, Tuzen M, Soylak M (2016). Flame atomic absorption spectrometric determination of gold after solid-phase extraction of its 2-aminobenzothiazole complex on Diaion SP-207. J AOAC Inter.

[19] Cadar O, Mocan T, Roman C, Senila M (2021). Analytical performance and validation of a reliable method based on graphite furnace atomic absorption spectrometry for the determination of gold nanoparticles in biological tissues. Nanomaterials.

[20] Konečná M, Komárek J (2007). Utilization of electrodeposition for electrothermal atomic absorption spectrometry determination of gold. Spectrochim Acta Part B At Spectrosc.

[21] Balaram V, Vummiti D, Roy P, Taylor C, Kar P, Raju AK, Abburi K (2013). Determination of precious metals in rocks and ores by microwave plasma-atomic emission spectrometry for geochemical prospecting studies. Curr Sci..

[22] Chen S, Yan J, Wang C, Lu D (2019). Preconcentration and determination of Au (III), Pd (II), and Pt (IV) using dispersive micro-solid phase extraction with multi-porous ZnFe2O4 nanotubes and ICP-MS. At Spectrosc.

[23] Fox J, Newham G, Bushby RJ, Valleley EMA, Coletta PL, Evans SD (2023). Spectrophotometric analysis and optimization of 2D gold nanosheet formation. J Phys Chem C.

[24] Khlebtsov NG, Khlebtsov BN, Kryuchkova EV, Zarkov SV, Burov AM (2022). Universal determination of gold concentration in colloids with UV–vis spectroscopy. J Phys Chem C.

[25] Hajinia A, Heidari T (2021). Sensitive fluorometric determination of gold in geological samples using fire assay pre-concentration coupled with microfluidic paper-based analytical device. Microchem J.

[26] García MBG, García AC (1995). Adsorptive stripping voltammetric behaviour of colloidal gold and immunogold on carbon paste electrode. Bioelectrochem Bioenerg.

[27] Caporali S, Bellandi S, Romualdi L, Bernardi S, Innocenti M, Pezzatini G (2015). Simultaneous determination of gold and palladium via potentiometric titration. Curr Anal Chem.

[28] Hamidatou LA (2019). Advanced technologies and applications of neutron activation analysis.

[29] Berhanu AL, Gaurav, Mohiuddin I, Malik AK, Aulakh JS, Kumar V, Kim KH (2019). A review of the applications of Schiff bases as optical chemical sensors. TrAC - Trends Anal Chem.

[30] Alshehri RF, Hemdan M, Babalghith AO, Amin AS, Darwish ER (2024). An innovative approach in titanium determination based on incorporating 2-amino-4-((4-nitrophenyl) diazenyl) pyridine-3-ol in a PVC membrane. RSC Adv.

[31] Shamsipur M, Poursaberi T, Karami AR, Hosseini M, Momeni A, Alizadeh N, Yousefi M, Ganjali MR (2004). Development of a new fluorimetric bulk optode membrane based on 2, 5-thiophenylbis (5-tert-butyl-1, 3-benzexazole) for nickel (II) ions. Anal Chim Acta.

[32] Kuswandi B, Vaughan AA, Narayanaswamy R (2001). Simple regression model using an optode for the simultaneous determination of zinc and cadmium mixtures in aqueous samples. Anal Sci.

[33] Glenn SJ, Cullum BM, Nair RB, Nivens DA, Murphy CJ, Angel SM (2001). Lifetime-based fiber-optic water sensor using a luminescent complex in a lithium-treated Nafion™ membrane. Anal Chim Acta.

[34] Sotomayor PT, Raimundo IM, Zarbin AJG, Rohwedder JJR, Neto GO (2001). Alves OL Construction and evaluation of an optical pH sensor based on polyaniline–porous Vycor glass nanocomposite. Sensors Actuators B Chem.

[35] Albero MI, Ortuno JA, Garcia MS, Cuartero M, Alcaraz MC (2010). Novel flow-through bulk optode for spectrophotometric determination of lithium in pharmaceuticals and saliva. Sensors Actuators B Chem.

[36] Gholivand MB, Niroomandi P, Yari A, Joshagani M (2005). Characterization of an optical copper sensor based on N, N′-bis (salycilidene)-1, 2-phenylenediamine. Anal Chim Acta.

[37] Ganjali MR, Zare-Dorabei R, Norouzi P (2009). Design and construction of a novel optical sensor for determination of trace amounts of dysprosium ion. Sensors Actuators B Chem.

[38] El-Feky HH, El-Bahy SM, Hassan AME, Amin AS (2023). Utility of a novel optical sensor design for ultra-trace detection of chromium colorimetrically in real environmental samples. Int J Environ Anal Chem.

[39] Moustafa IMI, Amin AS, Darwish E (2023). A novel bulk optode for ultra-trace detection of antimony coupled with spectrophotometry in food and environmental samples. Talanta Open.

[40] Amin AS, El-Bahy SM, Hassan AME (2023). Construction of an optical sensor for molybdenum determination based on a new ionophore immobilized on a polymer membrane. J King Saud Univ - Sci.

[41] Alshehri RF, Amin AS, Darwish ER (2023). Ultrasensitive and highly selective detection of nickel ion by two novel optical sensors. Anal Bioanal Chem..

[42] El-Feky HH, Amin AS, Moustafa EMI (2022). Utilization of a plasticized PVC optical sensor for the selective and efficient detection of cobalt (II) in environmental samples. RSC Adv.

[43] Moustafa EMI, Amin AS, El-Attar MA (2022). A highly selective bulk optode based on 6-{4-(2, 4-dihydroxy-phenyl) diazenyl) phenyl}-2-oxo-4-phenyl-1, 2-dihydro-pyridine-3-carbonitrile incorporating chromoionophore V for determination of nano levels of cadmium. Anal Biochem.

[44] Gouda AA, Amin AS (2022). Design of a novel optical sensor for determination of trace amounts of tin in food and in environmental samples. Int J Environ Anal Chem.

[45] Amin AS, El-Bahy S, El-Feky HH (2022). Utility of 5-(2′,4′-dimethylphenylazo)-6-hydroxy-pyrimidine-2,4-dione in PVC membrane for a novel green optical chemical sensor to detect zinc ion in environmental samples. Anal Biochem.

[46] Dalziel JAW. A text-book of quantitative inorganic analysis, including elementary instrumental analysis. A. I. Vogel: Third Edition. Pp xxx + 1216. Longmans, Green, London, 1961. 70s. J Inorg Nucl Chem. 1962;24:1300

[47] Moustafa ME, Mabrouk EM, Dessouki HA, Amine AS (1991). Spectrophotometric Microdetermination of copper (II), silver (I), and gold (III) using azastyrene schiff bases. Microchem J.

[48] Britton HTS. Hydrogen Ions. 4th ed, 2 vols. 1956.

[49] Soylak M, Colak H, Tuzen M, Turkoglu O, Elci L (2006). Comparison of digestion procedures on commercial powdered soup samples for the determination of trace metal contents by atomic absorption spectrometry. J Food Drug Anal.

[50] Sheng PP, Etsell TH (2007). Recovery of gold from computer circuit board scrap using aqua regia. Waste Manag Res.

[51] Alizadeh N, Moemeni A, Shamsipur M (2002). Poly (vinyl chloride)-membrane ion-selective bulk optode based on 1, 10-dibenzyl-1, 10-diaza-18-crown-6 and 1-(2-pyridylazo)-2-naphthol for Cu2+ and Pb2+ ions. Anal Chim Acta.

[52] Tavakkoli N, Shamsipur M (1996). Lead-selective membrane electrode based on dibenzopyrydino-18-crown-6. Anal Lett.

[53] Seiler K, Simon W (1992). Theoretical aspects of bulk optode membranes. Anal Chim Acta.

[54] Saidi K, Chaabani W, Dammak M (2021). Highly sensitive optical temperature sensing based on pump-power-dependent upconversion luminescence in LiZnPO 4: Yb 3+–Er 3+/Ho 3+ phosphors. RSC Adv.

[55] Ahmad M, Narayanaswamy R (2002). Optical fibre Al (III) sensor based on solid surface fluorescence measurement. Sensors Actuators B Chem.

[56] Li C-C, Kuo M-S (2002). Application of the acetylacetone chelation solid-phase extraction method to measurements of trace amounts of beryllium in human hair by GFAAS. Anal Sci.

[57] Hibbert DB, Thordarson P (2016). The death of the Job plot, transparency, open science and online tools, uncertainty estimation methods and other developments in supramolecular chemistry data analysis. Chem Commun.

[58] Baezzat MR, Karimi M (2013). Design and evaluation of a new optode based on immobilization of indophenol on triacetylcellulose membrane for determination of nickel. Int J ChemTech Res.

[59] Sanchez-Pedreno C, Ortuno JA, Albero MI, Garcia MS, Valero MV (2004). Development of a new bulk optode membrane for the determination of mercury (II). Anal Chim Acta.

[60] Tavallali H, Yazdandoust M (2008). Design and Evaluation of a mercury (II) optode based on immobilization of 1-(2-Pyridylazo)-2-Naphthol on a triacetylcellulose membrane and determination in various samples. Eurasian J Anal Chem..

